# Insight into the draft whole-genome sequence of the dermatophyte *Arthroderma vanbreuseghemii*

**DOI:** 10.1038/s41598-018-33505-9

**Published:** 2018-10-11

**Authors:** Mohamed Mahdi Alshahni, Tsuyoshi Yamada, Ayaka Yo, Somay Y. Murayama, Makoto Kuroda, Yasutaka Hoshino, Jun Ishikawa, Shinichi Watanabe, Koichi Makimura

**Affiliations:** 1 Institute of Medical Mycology, Teikyo University, 2-11-1, Kaga, Itabashi, Tokyo, 173-8605 Japan; 2 Teikyo University Institute of Medical Mycology, 359 Otsuka, Hachioji, Tokyo, 192-0395 Japan; 3 General Medical Education and Research Center, Teikyo University, 2-11-1, Kaga, Itabashi, Tokyo, 173-8605 Japan; 4 Laboratory of Medical Microbiology, School of Pharamcy, Nihon University, Narashinodai 7-7-1, Funabashi, Chiba, 274-8555 Japan; 5 Pathogen Genomics Center, National Institute of Infectious Diseases, Toyama 1-23-1, Shinjuku, Tokyo, 162-8640 Japan; 6 Department of Bioactive Molecules, National Institute of Infectious Diseases, Toyama 1-23-1, Shinjuku, Tokyo, 162-8640 Japan

## Abstract

Next-generation technologies have prompted efforts towards generating a large repertoire of whole-genome sequences. The dermatophyte *Arthroderma vanbreuseghemii* has been considered as a good model in which to conduct molecular biological studies on this fungal group. Despite the considerable repertoire of molecular tools developed for this fungus, the lack of genomic data has represented a major limitation, preventing effective implementation of those tools. Herein, the authors report the first draft whole-genome sequence of this dermatophytic species. The size of the draft genome was 23 Mb, exhibiting a GC content of 48.1%. Given the significance of secreted proteases in tissue invasion, a comparative analysis of genes encoding extracellular proteases was performed between *A*. *vanbreuseghemii* and other dermatophytes. Furthermore, genes that might be involved in DNA repair also were compared among dermatophytes. Moreover, the complete mitochondrial genome of *A*. *vanbreuseghemii* was obtained and shown to consist of 24,287 bp with a GC content of 24%. In conclusion, the availability of genomic data for *A*. *vanbreuseghemii* is expected to facilitate the implementation of the molecular tools established for this fungus, enhancing our understanding of the biology of dermatophytes.

## Introduction

Dermatophytes are fungal agents that invade the outer layers of the skin. Infections by this group are wide-spread around the world and are known to worsen the quality of human and animal lives while imposing a large economic burden for therapy^1–3^. Understanding the molecular biology of these fungi is expected to pave the way toward novel therapeutic options, a process that has been hampered by the lack of genomic data. However, recent advancements in next-generation technologies have permitted the sequencing of the genomes of many living organisms.

Draft genome sequences of several dermatophytes have recently been made available^4–6^, including that of *Trichophyton rubrum*. Whereas *T*. *rubrum* is the most commonly encountered dermatophytic pathogen, genetic manipulation studies are restricted in this species due to the frequent loss of its ability to sporulate. On the other hand, the zoophilic dermatophyte *Arthroderma vanbreuseghemii* (Syn. *Trichophyton mentagrophytes*^7^) strain TIMM 2789 is a species that is pathogenic in both human and animals and has been shown to undergo various genetic recombination processes without typically losing the ability to sporulate^8,9^. Accordingly, strain TIMM 2789 can be used as a model organism for the study of dermatophytes. This strain has been widely employed in genetic research, particularly in studies aimed at developing essential molecular tools. These tools include transformation methods, selectable markers, and a marker-recycling system, as well as the generation of TIMM 2789 derivatives with improved homologous recombination (HR) efficiencies as a result of disruption of the strain’s non-homologous end-joining (NHEJ) pathway^10–15^. However, application of these molecular tools has long been hampered by a lack of genomic data. Accordingly, the availability of whole-genome sequence for this strain is expected to ensure an optimum use of the already established molecular tools and to assist in efforts aimed at unveiling this dermatophyte’s pathogenicity.

Herein, the authors report the first (to our knowledge) draft genome sequence of the dermatophyte *A*. *vanbreuseghemii* and its complete mitochondrial genome (mtDNA) sequence. The authors also conducted a comparative study based on exploring several gene families and pathways that are of biological or medical importance.

## Results and Discussion

Draft genome sequences of some dermatophytic species have recently been made available^4–6^. However, most of these sequenced organisms lack molecular tools that would permit full application of these data; notably, many of these dermatophytes rapidly lose their ability to sporulate. In contrast, *A*. *vanbreuseghemii* strain TIMM 2789 is able to infect both humans and animals^9^, suggesting that TIMM 2789 might be a good model for the medical and veterinary study of pathogenic dermatophytes. Moreover, this strain has been the focus of considerable developments aimed at creating the platform required to conduct genetic studies^10–15^. However, the scarcity of genomic data on this strain has precluded the effective exploitation of these molecular tools. Collectively, these facts were the drive behind generating a draft nuclear and complete mtDNA genome sequences for *A*. *vanbreuseghemii* TIMM 2789.

### General features

**Figure 1:**
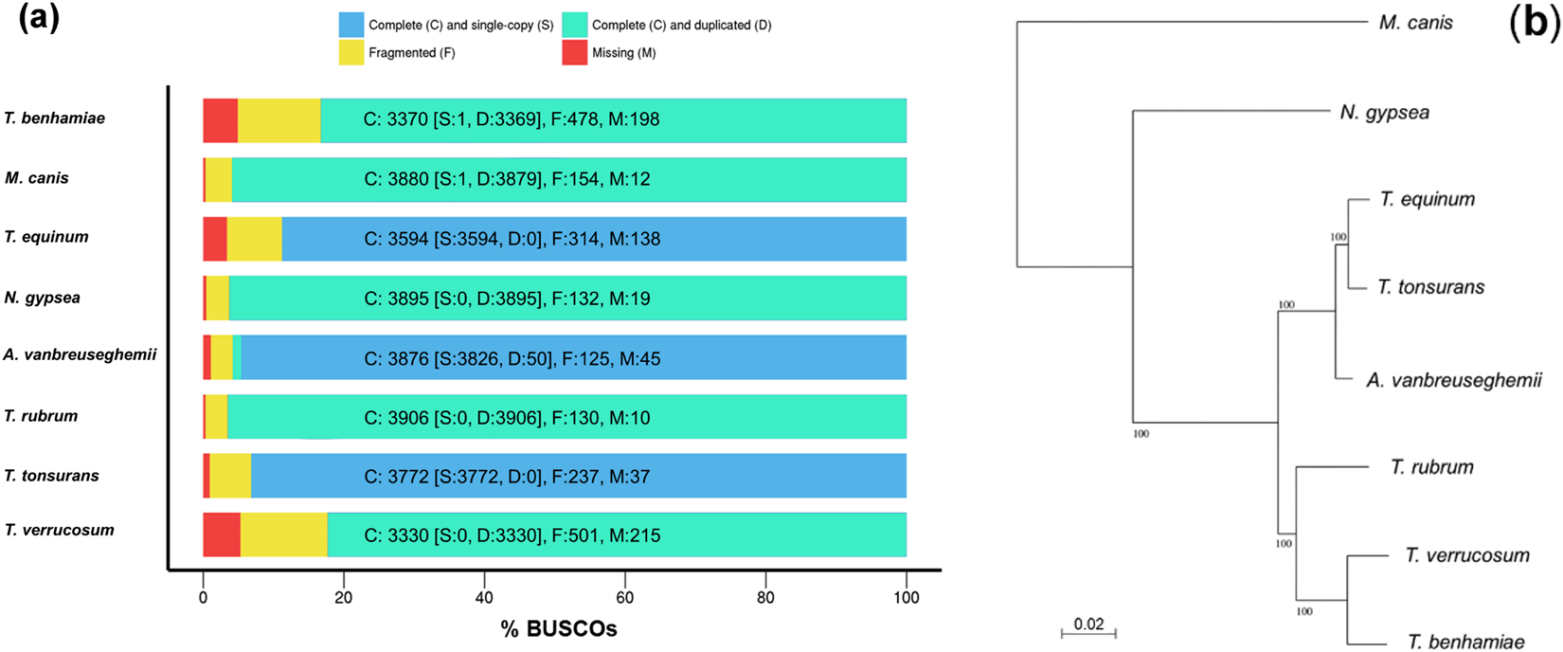
Evaluation of the quality of genome assembly of *A*. *vanbreuseghemii*. (**a**) Core gene assessment of the draft genome of *A*. *vanbreuseghemii* using a total of 4046 core genes. (**b**) Maximum likelihood-based phylogenetic analyses using concatenated 4,419 Orthologous protein families that were found common in the eight dermatophytic species. Numbers on branches are bootstrap percentages. Bar, 2 substitutions per 100 nucleotide positions.

The total number of reads was 50,179,254 with an average read length of 80 bp. The size of the draft whole-genome sequence was 23 Mb with a GC content of 48.1%; these values were similar to those obtained in the sequencing of other dermatophytic species. The sequences of *A*. *vanbreuseghemii* were assembled into contigs, the longest of which was 571.4 Kb. The assembly statistics yielded an N50 of 81.08 Kb, that is, half of the contigs exceeded 81 Kb in size. The sequences were assembled into 533 contigs with lengths ≥1000 bp. Based on the assumption that protein-coding genes were 30 codons or longer, a self-trained algorithm returned 7,860 genes, while algorithms trained using the *Coccidioides immitis* and *Histoplasma capsulatum* genomes returned 7,077 and 7,350 genes, respectively. However, these values may not represent the exact gene count, given the fragmented (multi-contig) nature of our draft genome assembly; though the core gene analysis showed that quality of the assembly is sufficient for subsequent analyses (Fig. 1a). In addition, phylogenetic analysis of the 4,419 orthologous protein families that were found common in eight dermatophytic species showed they are very closely related (Fig. 1b). Analyses for tRNA genes detected 101 genes (see Supplementary Table S1).

The mitochondrial genome of *A*. *vanbreuseghemii* strain TIMM 2789 is 24,287 bp in length. This value is 10 bp shorter than that for the mtDNA of *T*. *mentagrophytes* BMU03104, although the two mtDNAs exhibit a sequence similarity of 99.99%. In contrast to other dermatophytes that possess 25 mitochondrial tRNA genes^16^, TIMM 2789 has only 24 mitochondrial tRNA genes, reflecting a single nucleotide substitution in the TIMM 2789 sequence corresponding to the mitochondrial tRNA^Ser(GCT)^ in other dermatophytes. The overall GC content of the TIMM 2789 mtDNA is 24%.

**Figure 2:**
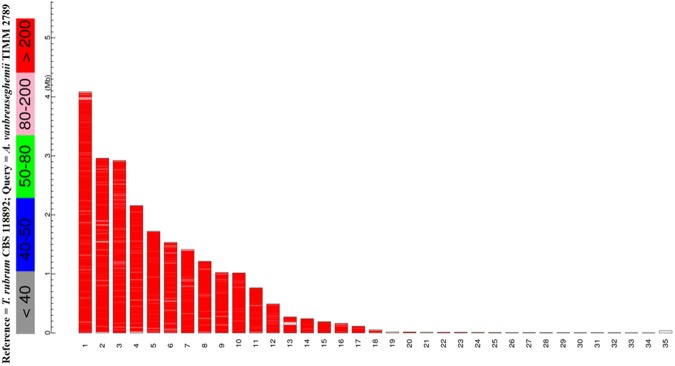
Megablast graphical overview of *A*. *vanbreuseghemii* vs. *T*. *rubrum*. Diagram indicates the hits of *T*. *rubrum* (reference) sequence aligned to *A*. *vanbreuseghemii* (query) sequence. Color key for the alignment scores is displayed at the top.

Genome sequence comparison to *T*. *rubrum* showed high similarity (Fig. 2, Supplementary Fig. S1 and Table 1), with an average nucleotide identity (ANI) of approximately 90%, implying that TIMM 2789 might be useful as a model dermatophyte, given the limitations of genetic recombination in *T*. *rubrum*. Notably, the draft genome of TIMM 2789 has lower similarity to *Microsporum canis* (a zoophilic fungus) than to *Nannizzia gypsea* (a geophilic fungus) (Table 1 and Supplementary Figs S2 and S3). Comparisons of the *A*. *vanbreuseghemii* nuclear genome to those of related species revealed the presence of 5879, 5854, 6138, 6143, 6231, 6140, and 6006 putative orthologues of the genes of *Trichophyton benhamiae*, *Trichophyton verrucosum*, *Trichophyton tonsurans*, *Trichophyton equinum*, *T*. *rubrum*, *N*. *gypsea*, and *M*. *canis*, respectively. The disparity in the number of orthologues may explain the host and site specificity of dermatophytes when invading various keratinous tissues of human and animals.

**Table 1:** Average nucleotide identity (ANI) of four dermatophytic species.

		Reference			
		*T*. *rubrum*	*A*. *vanbreuseghemii*	*N*. *gypsea*	*M*. *canis*
Query	*T*. *rubrum*	—	89.96%	80.74%	76.47%
	*A*. *vanbreuseghemii*	89.95%	—	80.94%	76.70%
	*N*. *gypsea*	80.73%	80.91%	—	77.02%
	*M*. *canis*	76.38%	76.56%	76.91%	—

### Analysis of extracellular proteases

**Figure 3:**
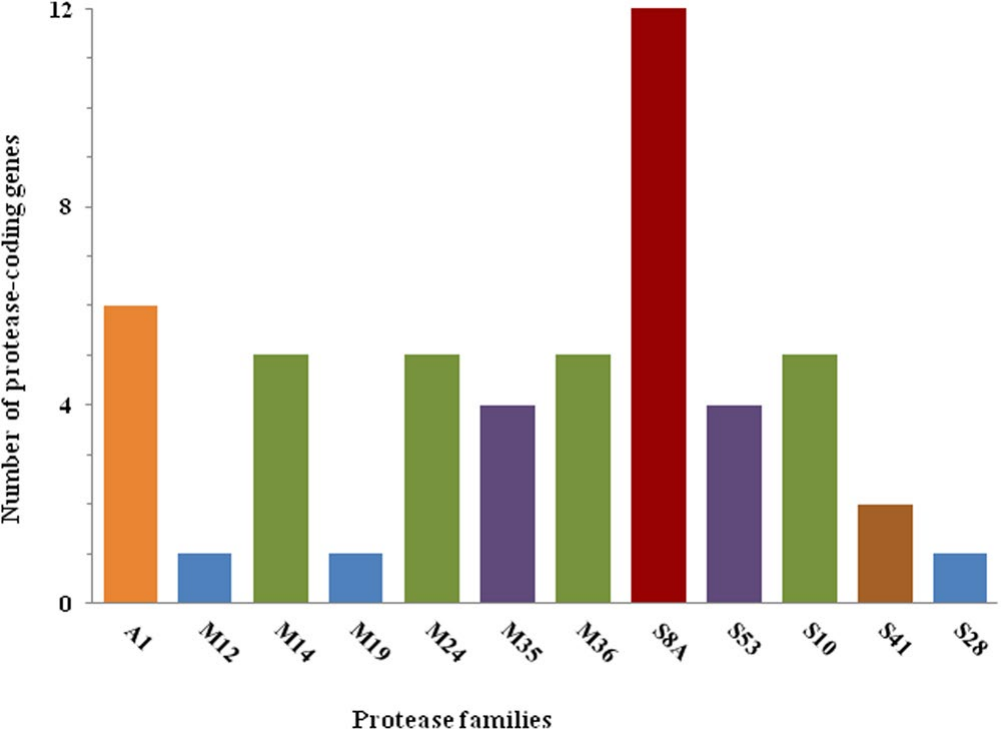
Major protease gene families predicted in *A*. *vanbreuseghemii*.

Dermatophytes possess a large repertoire of secreted proteases. A recent comparative genomic analysis demonstrated that dermatophyte proteomes are enriched for proteases^6^. Two major families of extracellular endoproteases are involved in the proteolytic activities of dermatophytes: subtilisin (family S8A)^17^ and fungalysin (family M36)^18^. In a previous study, we demonstrated that the proteolytic activity of TIMM 2789 in skimmed milk is primarily the result of the activity of metalloprotease-4 (MEP4)^15^. Although multiple knockout mutants have been generated, including strains mutated in the genes encoding the subtilisin-3 (SUB3), SUB6, and SUB7 proteins, the proteolytic activity of these mutants did not appear to be significantly altered compared to that of the parent^15^. In contrast, a MEP4-deficient strain exhibited impaired proteolytic activity. This result is consistent with the effect of metalloprotease inhibition using phosphoramidon. Analyses of the draft genome sequences of TIMM 2789 predicted 12 SUB-encoding genes and 5 MEP-encoding genes (see Supplementary Table S2). The number of copies of those gene families is similar to the numbers in the majority of other sequenced dermatophytes, with the exception of *T*. *tonsurans* and *T*. *rubrum*. On the other hand, TIMM 2789 has fewer deutrolysin (M35) -encoding genes than other sequenced dermatophytes (4 genes in TIMM 2789 vs. 5–6 in other sequenced species^6^). A summary of the distribution of the major protease gene families in these fungi is provided in Fig. 3.

### DNA repair analysis

DNA repair is a natural defense mechanism to protect genomic information from damaging factors. Repair of DNA double-strand breaks (DSBs) occurs through either the HR or NHEJ pathways. As in other filamentous fungi, DSBs are repaired predominantly through NHEJ in TIMM 2789^13^. In addition to its significance in preserving genetic data, the DNA repair process plays a key role in gene-targeting studies aimed at exploring the function(s) of individual genes. In dermatophytes, DNA repair mechanisms are largely unknown. Attempts aimed at improving gene-targeting frequencies in TIMM 2789 were made by producing *Ku80* and *Lig4* null mutants^11,13^. While the KU70-KU80 heterodimer initiates DSB repair by binding to broken DNA ends, Lig4 functions at the final stage of the NHEJ pathway by forming a complex through joining of Xrcc4 proteins via the BRAC1 domains. The *Ku80* and *Lig4* single mutant strains exhibited enhanced gene-targeting efficiencies compared to the wild-type parent. However, unlike the case in other filamentous fungi (e.g., *Aspergillus oryzae*), impairment of NHEJ in TIMM 2789 did not lead to HR frequencies as high as 100%^19^, suggesting a distinct mechanism of HR regulation in *A*. *vanbreuseghemii*. Notably, our comparative genomic analysis revealed that TIMM 2789 is enriched (compared to other sequenced dermatophytes) for paralogues of the *RcaA* DNA damage response gene, a member of the forkhead associated domain (FHA) superfamily. While *A*. *vanbreuseghemii* carries three *RcaA* paralogues on separate contigs, other dermatophytes possess only a single copy. Supplementary Table S3 lists putative genes that may be involved in DNA repair in *A*. *vanbreuseghemii*.

## Conclusion

This is the first report of the draft genome sequences of the dermatophyte *A*. *vanbreuseghemii*. Likewise other dermatophytes, the fungus is enriched with protease-encoding genes. Moreover, the number of orthologues varies among dermatophytes, which may explain the host and site specificity of these closely related group. The availability of genomic data for *A*. *vanbreuseghemii*, along with a long list of already established molecular tools, would allow more in-depth studies aimed at understanding dermatophytes.

## Material and Methods

### Strain and genomic DNA preparation

The *A*. *vanbreuseghemii* strain^9^ that was used for sequencing was TIMM 2789, which has also been designated as VUT 77007, SM 110, and RV 27960^T^. This strain is available from the Japan Collection of Microorganisms (JCM) as strain JCM 1891. TIMM 2789, mating-type (+), grows at standard rates, forms conidia normally, and can be used to generate protoplasts. Total DNA was prepared by growing the strain on Sabouraud dextrose agar (SDA) for 3 days at 28 °C; total DNA was extracted according to the method described previously^20^. To remove RNA from the preparation, the extracted DNA was treated with ribonuclease A (RNaseA, Thermo Scientific).

### Sequencing, assembly, and annotation

The genome of TIMM 2789 was sequenced by the whole genome shotgun approach using an Illumina GAIIx platform (Illumina Inc., San Diego, CA, USA). A paired-end library was prepared from genomic DNA using the Nextera kit (Illumina Inc.), in accordance with the manufacturer instructions. The library was constructed to contain random inserts with an average size of 500 bp. TIMM 2789 was sequenced to 160-fold coverage. The sequence reads were deposited into the DNA Data Bank of Japan (DDBJ)’s Sequence Read Archive (DRA) under accession number DRA006383. The genome was assembled using ABySS version 1.3.2^21^ and annotated using GeneMark-ES^22^ for self-training and Augustus^23^ for training using *C*. *immitis* and *H*. *capsulatum*. Nuclear genome loci coding for tRNAs were identified using tRNAscan-SE^24^. Annotation was performed by combining together protein Basic Local Alignment Search Tool (BLASTP), Pfam^25^, InterProScan^26^ and orthologue analyses. Orthologues were identified as reciprocal best-hit pairs using the BLASTP program. The genomic similarity comparisons between *A*. *vanbreuseghemii* and other dermatophytes were performed using megablast ver. 2.2.26. BLAST-based ANI between each pair of genomes was calculated using JSpecies ver. 1.2.1^27^. The completeness of the draft genome assembly was assessed in term of core gene by BUSCO v3^28,29^ using 4046 core genes of Eurotiomycetes dataset (see Supplementary Table S4). Orthologous proteins among eight dermatophytic species (*A*. *vanbreuseghemii*, *T*. *benhamiae*, *T*. *equinum*, *T*. *rubrum*, *T*. *tonsurans*, *T*. *verrucosum*, *M*. *canis and N*. *gypsea*) were identified by bidirectional best blastp hits method. Individual families were aligned by ClustalW v2.1^30^, and concatenated by species. A phylogenetic tree was constructed according to maximum likelihood method using RAxML v7.3.4^31^.

### Detection of mitochondrial genome of TIMM 2789

The mtDNA of strain TIMM 2789 was identified by searching the draft TIMM 2789 genome for sequences with similarity to the mtDNA of *T*. *mentagrophytes* (human type; BMU03104)^16^. A locus with high similarity was detected. Internal primers were designed to fill the gaps in the locus in comparison with the mtDNA of BMU03104 (see Supplementary Table S5). Sequencing was performed with an ABI PRISM^®^ 3130xl Genetic Analyzer (Applied Biosystems, Carlsbad, CA, USA) using the BigDye^®^ Terminator Sequencing Kit (Applied Biosystems). DNA sequences were deposited in GenBank under accession number MG592681.

## Electronic supplementary material

Supplementary Information

Supplementary Dataset
