# Supplementary material for: Insight into the draft whole-genome sequence of the dermatophyte Arthroderma vanbreuseghemii
Source: Sci Rep. 2018 Oct 11;8:15127. doi: 10.1038/s41598-018-33505-9 (PMC6181936; doi:10.1038/s41598-018-33505-9)
Supplement: Supplementary file 1 — Supplementary Information [file 41598_2018_33505_MOESM1_ESM.pdf]

Insight into the draft whole-genome sequence of the dermatophyte *Arthroderma vanbreuseghemii*

Running title: Draft genome sequence of *A. vanbreuseghemii*

Keywords: dermatophytes; genome; mitochondria; DNA repair; protease.

Mohamed Mahdi Alshahni[1, 2, 3], Tsuyoshi Yamada[2, 3], Ayaka Yo[1], Somay Y. Murayama[4], Makoto Kuroda[5], Yasutaka Hoshino[6], Jun Ishikawa[6], Shinichi Watanabe[2], and Koichi Makimura[1, 2, 3]*

[1] Institute of Medical Mycology, Teikyo University, 2-11-1, Kaga, Itabashi, Tokyo 173-8605, Japan.

[2] Teikyo University Institute of Medical Mycology, 359 Otsuka, Hachioji, Tokyo 192-0395 Japan.

[3] General Medical Education and Research Center, 2-11-1, Kaga, Itabashi, Tokyo 173-8605, Japan.

[4] Laboratory of Medical Microbiology, School of Pharamcy, Nihon University, Narashinodai 7-7-1, Funabashi, Chiba 274-8555, Japan.

[5] Pathogen Genomics Center, National Insititute of Infectious Diseases, Toyama 1-23-1, Shinjuku-ku, Tokyo 162-8640, Japan.

[6] Department of Bioactive Molecules, National Institute of Infectious Diseases, Toyama 1-23-1, Shinjuku-ku, Tokyo 162-8640, Japan.

*Corresponding author. Mailing address: Institute of Medical Mycology, Teikyo University, 2-11-1, Kaga, Itabashi, Tokyo 173-8605, Japan.

Tel: +81-03-3964-8367   Fax: +81-03-3964-8413   E-mail: makimura@med.teikyo-u.ac.jp

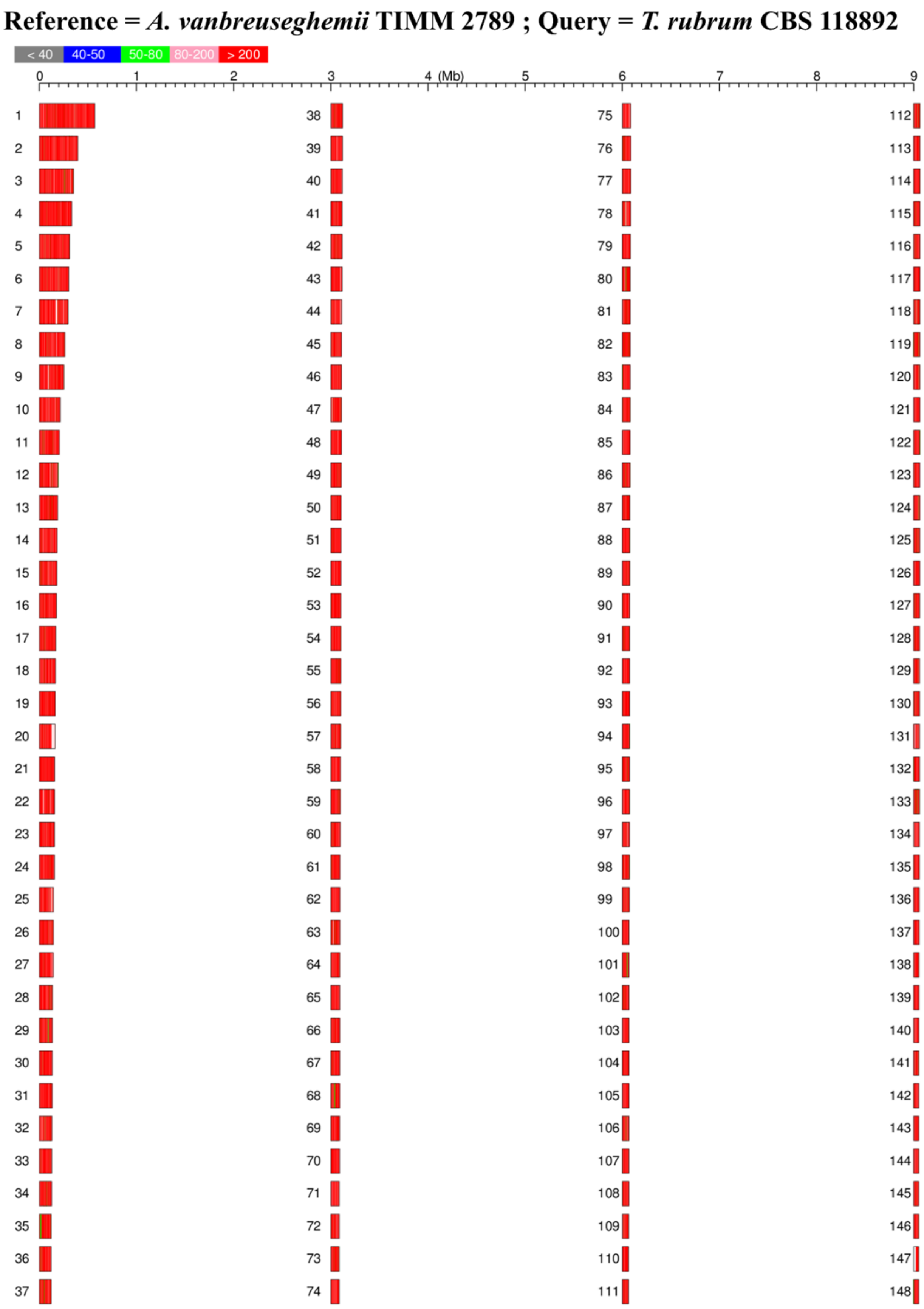

Fig. S1. Megablast graphical overview of *A. vanbreuseghemii* vs. *T. rubrum*. Diagram indicates the hits of *T. rubrum* (query) sequence aligned to *A. vanbreuseghemii* (reference) sequence. Color key for the alignment scores is displayed at the top.

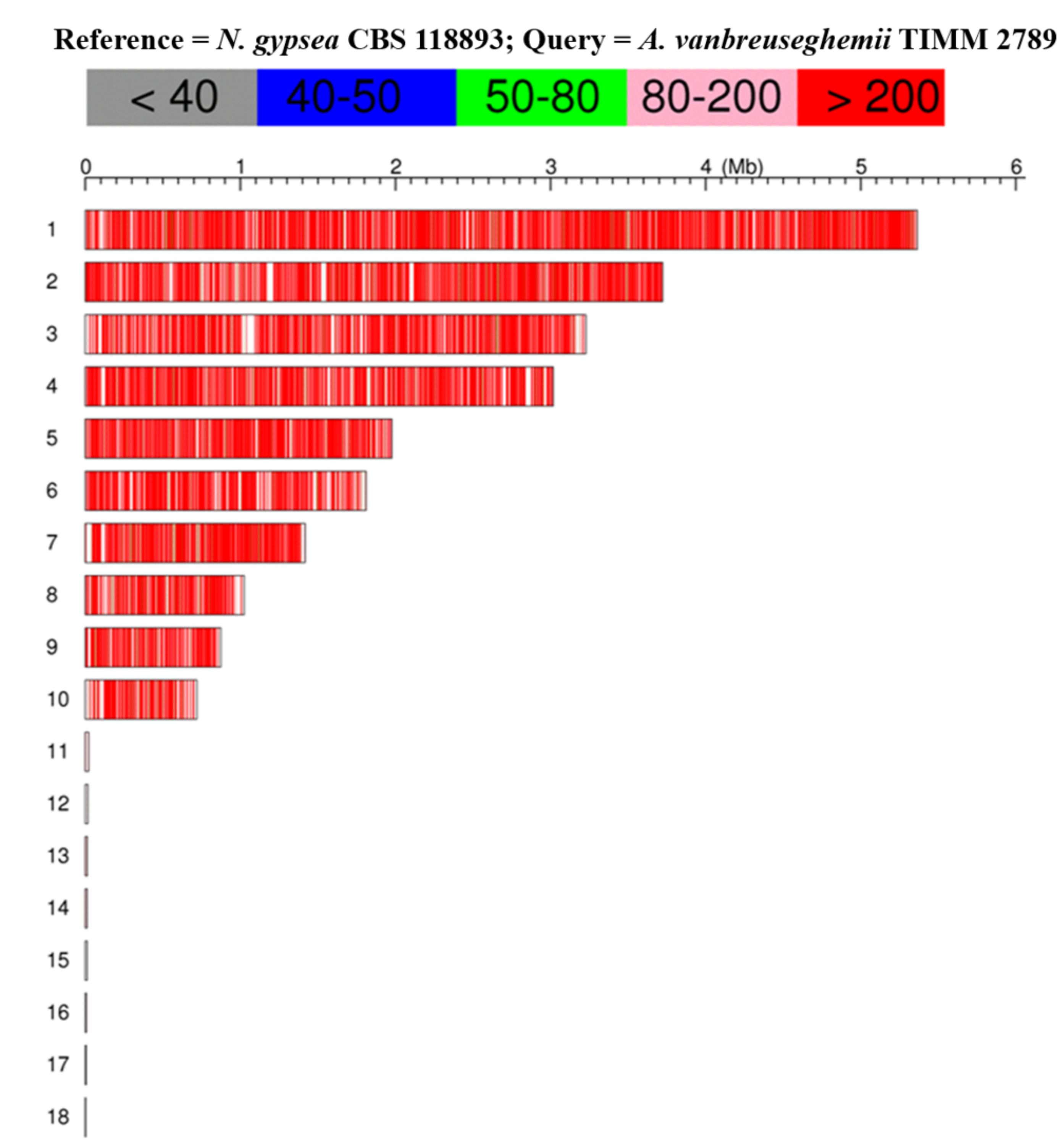

Fig. S2. Megablast graphical overview of *A. vanbreuseghemii* vs *N. gypsea*. Diagram indicates the hits of *N. gypsea* (reference) sequence aligned to *A. vanbreuseghemii* (query) sequence. Color key for the alignment scores is displayed at the top.

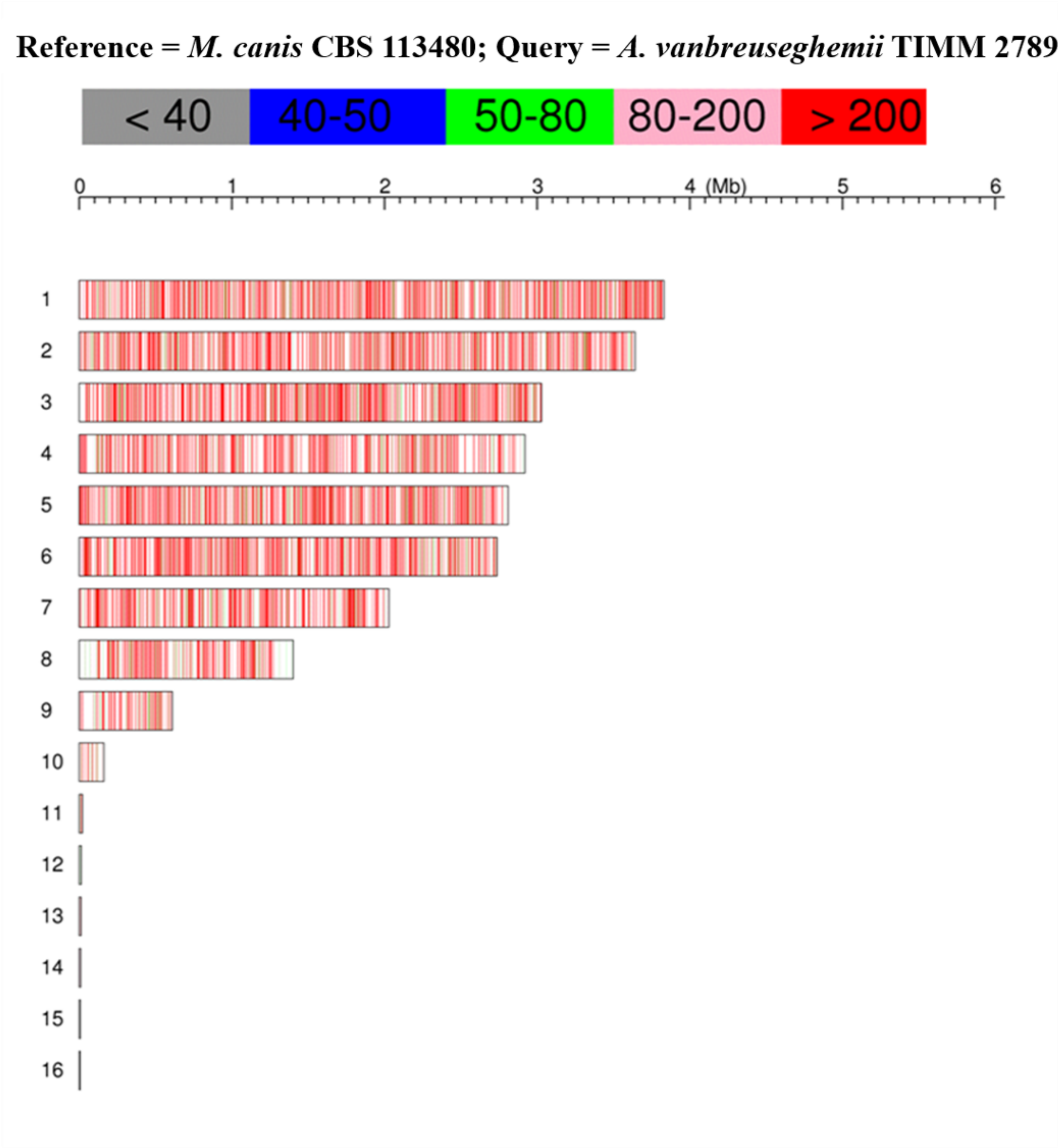

Fig. S3. Megablast graphical overview of *A. vanbreuseghemii* vs. *M. canis*. Diagram indicates the hits of *M. canis* (reference) sequence aligned to *A. vanbreuseghemii* (query) sequence. Color key for the alignment scores is displayed at the top.
